# Orbital Cellulitis in a Patient With Sphenoid Wing Meningioma

**DOI:** 10.7759/cureus.19323

**Published:** 2021-11-06

**Authors:** Thiruvarasu Gunasekaran, Kenneth Teow Kheng Leong, Pua Tze Hui

**Affiliations:** 1 Department of Ophthalmology, Hospital Seri Manjung, Sitiawan, MYS; 2 Department of Ophthalmology, Hospital Bintulu, Bintulu, MYS

**Keywords:** diagnostic dilemma, tumour lysis syndrome, proptosis, sphenoid wing meningioma, orbital cellulitis

## Abstract

We report a case of a 43-year-old woman with an underlying right sphenoid wing meningioma (SWM) who complained of increased right eye swelling, proptosis, redness and severe pain for two weeks. Her symptoms started one week after completing radiotherapy. She seeked treatment after a worsening of symptoms. An urgent computed tomography (CT) scan of the brain was done and showed increasing extension of tumour and hypodense areas within intraorbital region of the tumour with intraorbital fat stranding. She was treated as right eye orbital cellulitis with a differential diagnosis of tumour lysis syndrome. She was started on a combination of intravenous antibiotics and improvements were noticed after two days of treatment. This report presents the diagnostic challenge in managing orbital swelling in a patient with sphenoid wing meningioma with inconclusive radiological findings. Orbital cellulitis is an ocular emergency that requires prompt treatment and can potentially be vision and life-threatening, if not addressed early. In such cases with diagnostic dilemma, the decision to treat should be made as early as possible.

## Introduction

Orbital cellulitis is an ophthalmic emergency caused by infection of tissues, posterior to the orbital septum. The paranasal sinuses, especially the ethmoidal sinuses are the most common sources of infection which can result in orbital cellulitis [1]. Other sources include infection from adjacent ocular adnexal structures, periorbital structures and via exogenous routes such as traumatic injury. Infection can also spread through distant hematogenous routes, upper respiratory tract infection, dental infection, retained foreign bodies and surgeries [1]. In this report, we present a rare case of orbital cellulitis in a middle-aged woman with underlying sphenoid wing meningioma (SWM).

## Case presentation

**Figure 1:**
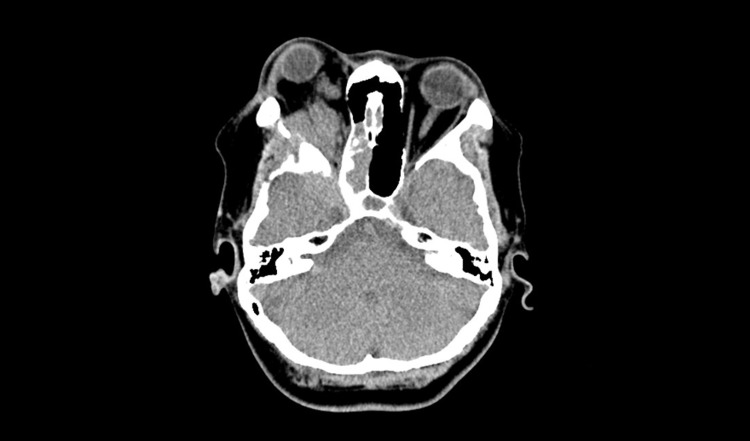
Axial post contrast computed tomography image demonstrates sphenoid wing meningioma with extension into right optic canal, right posterior ethmoid and sphenoid sinuses posteriorly. Captured seven months prior to third surgery.

This patient is a 43-year-old woman with an underlying history of right SWM with intraorbital extension, first diagnosed in 2014. She underwent a right craniotomy and tumour excision (Simpson II) in the same year but repeated imaging showed residual tumour over right sphenoid and orbital wall. Initial histopathological findings confirmed the diagnosis of meningothelial meningioma WHO Grade 1. Due to tumour progression, she had a severe right eye (RE) proptosis and a right orbital decompression was done by the Neurosurgical team in 2015. Subsequently, in 2016 her tumour continued to progress with involvement of right posterior ethmoid and sphenoid sinuses and she underwent radiotherapy. She was referred for eye assessment and noted to have RE proptosis, restricted extraocular movement and vision was perception to light. She subsequently defaulted follow-up. The surgical team continued to monitor her tumour progression with a six-monthly imaging until 2019 when her MRI brain showed evidence of increased size of residual mass with new extension into right temporal region, as well as another new right temporal mass. A third surgery involving craniectomy and debulking was done. Two months later, she was treated with radiotherapy to a total dose of 60Gy given in 30 fractions (Figure 1).

**Figure 2:**
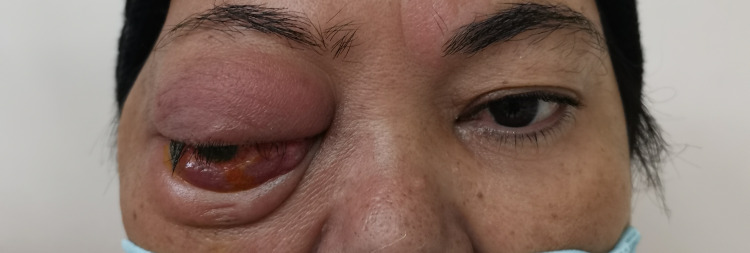
Photograph taken at two weeks of presentation showing patient's right-sided orbital cellulitis with worsening proptosis, ophthalmoplegia and chemosis. Lagophthalmos is also seen when closing the right eye, causing exposure of the inferior conjunctiva.

Three weeks after completing radiotherapy she complained of worsening of RE proptosis for one-week duration, associated with pain with a score of 3/10. She had no fever or any other symptoms. On examination, her RE vision was non-perception to light (NPL), positive relative afferent pupillary defect and no ocular motility. She had corneal epithelial defect and lagophthalmos due to proptosis, conjunctival hyperemia and chemosis. Her fundus examination showed a pale disc but not swollen. Left eye (LE) vision was 6/7.5 and ocular findings were unremarkable. Neurological examination and review of general systems showed normal findings. She was diagnosed with exposure keratopathy and managed conservatively with close monitoring of her proptosis. A week later, the patient returned with fever and rapid worsening of RE proptosis, redness and pain, as can be seen in Figure 2.

**Figure 3:**
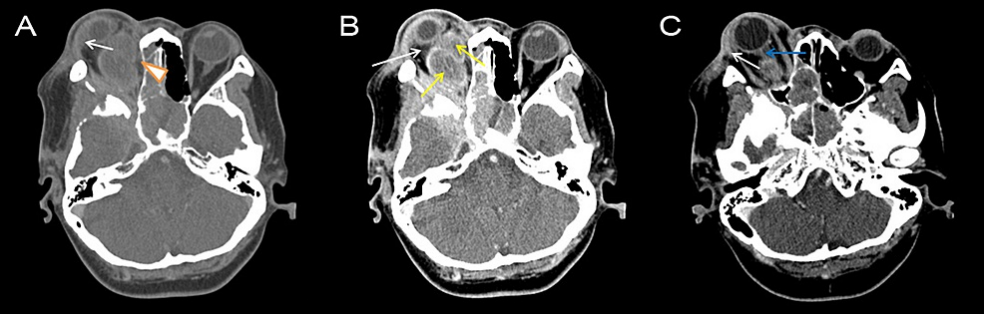
Axial post contrast computed tomography image demonstrates intraorbital extension of sphenoid wing meningioma with intraorbital fat stranding (white arrows), right optic nerve thickening (blue arrow) and two oval hypodense regions with rim enhancement, representing collections (yellow arrows). Arrowhead shows the location of possible right subperiosteal abscess.

An urgent CT orbit and brain was done (Figure 3A-3C) and there was evidence of increasing size of the residual mass along with two hypodense regions with rim enhancement within the right orbital extended mass. There was also thickening of the right optic nerve and increasing degree of intraorbital fat stranding suggesting possible infection and/or tumour lysis syndrome. Baseline blood investigations were performed and full blood count showed leucocytosis with neutrophilia. Blood culture however reported the absence of microorganisms. The patient also refused surgical biopsy which was offered to her. 

She was treated as right orbital cellulitis and was immediately commenced on intravenous (IV) metronidazole 500mg TDS and IV ceftriaxone 2 grams OD. Two days later, her right eye proptosis and other symptoms were markedly reduced. IV dexamethasone 8mg TDS was commenced on the third day following improvement of symptoms and completed for three days. The IV antibiotics therapy was continued for a total of two weeks. Final review on day 14 showed remarkable improvement. Patient was able to fully close her right eye and pain-free. Patient was subsequently discharged however her RE vision remained NPL.

## Discussion

Meningioma is a tumour that arises from meninges and constitutes about 20% of all intracranial tumors [2]. This includes sphenoid wing meningioma, also known as spheno orbital meningioma (SOM). SOMs are secondary tumours of the orbit that exhibit slow growing nature [3]. Due to the sphenoid wing’s close proximity with vital structures such as cranial nerves, carotid artery and cavernous sinus, the SOM requires periodic ophthalmological evaluation due to its compressive effect on these structures, which may eventually manifest with symptoms such as opthalmoplegia, proptosis and decreased visual acuity, as seen in our patient.

The challenging aspect of our patient’s illness is that there are several possible differentials based on these symptoms and radiologic findings. The CT orbit of our patient demonstrated intraorbital fat stranding, right optic nerve thickening and the presence of intraorbital hypodense regions, resembling collections. These collections could be infective or inflammatory, or may represent tumour lysis or post radiotherapy changes. Symptoms such as sudden onset with rapid progression of proptosis, pain and fever with high white cell count were suggestive of infection. There was uncertainty as some of the symptoms can be attributed to deterioration of the primary tumor or orbital cellulitis. However, primary working diagnosis of orbital cellulitis was made based on CT and clinical findings.

The acute and rapid progression of the swelling was not keeping with the slow-growing nature of SOM and raised a high suspicion of malignant transformation of the tumour. Withstanding, WHO classification remains the reliable grading of meningioma based on morphological features, benign (grade I), atypical (grade II) and malignant (grade III) [4]. Radiological findings such as peritumoural edema, ‘mushrooming’, bone involvement and tumour calcification are useful in predicting recurrences and offers little diagnostic value in distinguishing malignant from benign tumours [5]. However, a retrospective study proposed an association between atypical histology of meningioma with several findings, such as large tumour size and presence of necrotic collection, which was detected in the CT findings of this patient [6]. Thus the lack of recent histological documentation of the tumour does not completely rule out the possibility of a higher-grade meningioma.

A particular differential of interest is tumour lysis syndrome (TLS), which was formulated based on radiological findings of intraorbital collections in this patient. TLS is an oncologic emergency with a constellation of symptoms which is common in hematologic cancers. It can occur as a consequence of tumour targeted therapy such as chemotherapy and radiotherapy or spontaneously [7]. However, based on diagnostic proposal by Cairo and Bishop, this case does not meet any of the criteria to be defined as either laboratory or clinical TLS [8]. Although neurological solid tumors such as medulablastoma and neuroblastoma can result in TLS, there are no reported cases of TLS in meningiomas to date [8]. Furthermore, the radiosensitivity of meningiomas is controversial [9]. Whether meningiomas are adequately radiotherapy sensitive to be causing rapid destruction of large numbers of cancer cells, is unknown.

A difficult aspect of this case was not knowing the source of infection. One of the theories that was postulated is the immunosuppression following radiotherapy. The infection can be endogenous in origin. The breeding and translocation of bacterial flora may have happened from sites in which they reside in, including skin, gastrointestinal tract, conjunctiva and lungs. The exogenous source that can be identified is the postoperative site. However, the likeliness of an infection occurring three months after surgery, especially without specific symptoms of intracranial infection, is low. A surgical biopsy or drainage of orbital abscess is the definitive way to confirm whether there is an infection.

In such situation with diagnostic dilemma which requires prompt attention, it is important to make a decision to treat as soon as possible. Thus during the patient’s second visit, broadspectrum antibiotics were started immediately on admission to cover for orbital cellulitis. Commonly associated microorganisms in orbital cellulitis are *Staphylococcus aureus*, *Streptococcus* and *Haemophilus* species [1]. In this patient, Ceftriaxone which covers most gram-positive and gram-negative bacteria was given intravenously. As the source of infection is uncertain and no culture was obtained, Metronidazole was also initiated to cover additional anaerobic bacteria which is also common in dental infections.

A short duration of steroid therapy was given for this patient to hasten the resolution of inflammation and improve overall outcome. The role of steroids in the treatment of orbital cellulitis is unclear and has not been heavily studied. A study by Pushker et al. supported the use of steroids in orbital cellulitis by demonstrating earlier resolution of signs and symptoms with the use of steroids adjunct to antibiotics therapy [10].

## Conclusions

Orbital cellulitis is an emergency that requires prompt attention and can potentially be vision and life threatening, if not addressed early. This case shows that rapid onset proptosis in a pre-existing intracranial tumour with orbital extension, can present with diagnostic challenge. In such cases, orbital cellulitis should always be included as one of the primary diagnosis. The diagnosis should be based on clinical symptoms, laboratory studies and characteristic neuroimaging findings. Appropriate choice of antibiotics with adequate duration with combination of steroid is required to achieve successful patient outcome.
